# The Mechanical Properties of Nanocomposites Reinforced with PA6 Electrospun Nanofibers

**DOI:** 10.3390/polym15030673

**Published:** 2023-01-28

**Authors:** Inga Lasenko, Jaymin Vrajlal Sanchaniya, Sai Pavan Kanukuntla, Yagnik Ladani, Arta Viluma-Gudmona, Olga Kononova, Vitalijs Lusis, Igors Tipans, Turs Selga

**Affiliations:** 1 Mechanics and Biotextile Research Laboratory, Riga Technical University, 3/3-20 Pulka Street, LV-1007 Riga, Latvia; 2 Department of Theoretical Mechanics and Strength of Materials, Institute of Mechanics and Mechanical Engineering, Riga Technical University, 6B Kipsala Street, LV-1048 Riga, Latvia; 3 Department of Molecular Biology, Faculty of Biology, Latvian University, Jelgavas Street 1, LV-1004 Riga, Latvia

**Keywords:** nanocomposite, multilayered composites, spun nanofiber, electrospinning

## Abstract

Electrospun nanofibers are very popular in polymer nanocomposites because they have a high aspect ratio, a large surface area, and good mechanical properties, which gives them a broad range of uses. The application of nonwoven structures of electrospun nanofiber mats has historically been limited to enhancing the interlaminar responses of fiber-reinforced composites. However, the potential of oriented nanofibers to improve the characteristics of bulk matrices cannot be overstated. In this research, a multilayered laminate composite was created by introducing polyamide (PA6)-oriented nanofibers into an epoxy matrix in order to examine the effect of the nanofibers on the tensile and thermal characteristics of the nanocomposite. The specimens’ fracture surfaces were examined using scanning electron microscopy (SEM). Using differential scanning calorimetry (DSC) analysis, the thermal characteristics of the nanofiber-layered composites were investigated. The results demonstrated a 10.58% peak in the nanocomposites’ elastic modulus, which was compared to the numerical simulation and the analytical model. This work proposes a technique for the development of lightweight high-performance nanocomposites.

## 1. Introduction

Polyamides, often known as nylons, are a category of synthetic polymers distinguished by their high strength, tensile strength, and abrasion resistance. They are formed of repeating units called amides that are connected by peptide bonds [1]. Polyamide electrospun nanofibers produced by electrospinning a polyamide polymer. Electrospinning involves applying a high-voltage electric field to a polymer solution or melt, causing the polymer to be extruded through a nozzle as a fine, continuous fiber. The high voltage also pulls and stretches the extruded polymer fibers, resulting in the creation of fibers with nanometer-scale diameters. In recent years, polyamide electrospun nanofibers have attracted significant interest due to their unusual features, which include a high surface area-to-volume ratio, high porosity, and excellent mechanical strength. These characteristics make them appropriate for a variety of applications, including filtration, tissue engineering, and drug delivery [2].

Epoxy resins are often used as matrix materials in composites because of their adhesiveness, chemical resistance, electrical insulation, light weight, and strong bonding qualities [3]. The addition of micro- or nanofillers to polymer materials has been used to achieve the desired mechanical and thermal characteristics [4,5,6,7,8]. Nanofiber-reinforced composite laminates can have a higher tensile strength [9], interlaminar fracture toughness [10,11], resistance to delamination [12,13] under static, impact, and fatigue loads. These characteristics depend on the polymer, chemical, physical, and mechanical parameters, as well as the additives. Nanofibrous reinforcement might be particularly beneficial for preventing delamination from any cause, including matrix fissures, notches, holes, and bolted joints [14,15]. The purpose of nanofibers is to bridge neighboring plies and minimize the stress concentration without compromising the in-plane characteristics [16]. Nanofibers may be used to strengthen the matrix between neighboring plies of a laminate [15,17]. There are several methods for producing nanofibers, including jet blowing, melt blowing, coextrusion, interfacial polymerization, and electrospinning [18,19,20]. Among these approaches, electrospinning has been extensively used to create polymer nanofibers, nanocomposites, as well as nanofibers containing different nanoparticles [21,22,23,24], graphene [25], graphene oxide [26], and carbon nanotubes [27] for different applications, such as biomedical [28,29], energy conversation [30], and tissue engineering [31,32]. Furthermore, the use of an electrospun thermoplastic nanofiber mats to toughen the glass/carbon epoxy laminated composites without degrading the in-plane mechanical properties and without considerably increasing the laminate thickness and weight has emerged as a promising technology [33,34]. The nanofibers have no negative influence on the epoxy resin reinforcement’s impregnation with various composites [35,36]. As a result, they may be applied to the conventional composite structure production procedures as a standalone product or as a fabric reinforcing layer.

Compared to more conventional materials such as metals and ceramics, polymer fiber-reinforced composites possess several unique characteristics, including a high stiffness and strength-to-weight ratio, excellent corrosion resistance, and the capacity to provide both mechanical and functional properties [37,38]. How to achieve high specific stiffness and strength is known. However, it is more difficult to adapt a fiber composite to combine an excellent mechanical performance and the required functional behavior needed, for instance, in transport, aerospace, energy, textiles, and healthcare [39]. There have been several efforts as discussed above to improve the mechanical characteristics of composites (CFRP and GFRP) by using electrospun nanofibers in laminates. Additionally, it is required to study the impact of nanofiber mats layered with simply epoxy, which might aid in the improvement of composites’ structural integrity. By mixing spun nanofibers with epoxy, the authors constructed laminated composites.

The orientation of fibers in composites is crucial because it dictates the direction of strength and stiffness of the fibers. If the fibers are aligned with the applied load, the composite material will be stronger and stiffer in that direction. If the fibers are aligned perpendicular to the applied load, however, the composite material will be weaker and less rigid in that direction. By changing the orientation of the fibers, the overall mechanical characteristics of a composite material may be adapted to meet the needs of a particular application. 

The structure and mechanical characteristics of nanofiber mats produced by electrospinning rely on a number of factors, including the collector type [40], rotating drum speed [41], collector distance, syringe diameter, and polymeric solution flow rate via the syringe [42]. Reducing the nanofiber’s diameter enhances its strength [21]. Therefore, it is essential to produce a nanofiber mat with uniformly oriented nanofibers in order to obtain the greatest performance from the nanocomposite created from this nanofiber mat. 

This article explores the use of nanofibrous polymeric layers to improve the mechanical characteristics, such as the tensile strength, of nanocomposites. Electrospinning was used to create nanofibers for PA6 layers, which were then encapsulated in an epoxy. Three distinct layers were reinforced to strengthen the epoxy (for a total of seven layers, including the epoxy). A tensile testing machine and a DSC was used to conduct a tensile test and a thermal test. The morphology of the nanofibers and the fracture surface was characterized using scanning electron microscopy after the tensile test. A numerical simulation was performed to compare the tensile test outcomes. 

## 2. Materials and Methods

### 2.1. Materials 

PA6 and formic acid were used to produce the electrospun nanofibers, and epoxy was used to make the laminated nanocomposites. PA6 (Polyamide 6, nylon 6, polycaprolactam; CAS: 25038-54-4, PA6 standard density: 1.06–1.16 g/cm^3^ (ISO 1183)) and formic acid, 99.0+%, Optima™ LC/MS Grade, Fisher Chemical™ (CAS: 64-18-6; Molecular Formula: CH2O2 Molecular Weight (g/mol): 46.025 MDL Number: 3297) were obtained from Sigma-Aldrich chemicals (Merck KGaA, Darmstadt, (64287) Germany). Liquid resin epoxy and hardener (CAS: 964-67-8) were obtained from the SIA “Latwood Master razosanas komercfirma” (Raunas, Str. 19, Riga, (LV1039) Latvia).

### 2.2. Fabrication of the Nanofibers 

**Figure 1:**
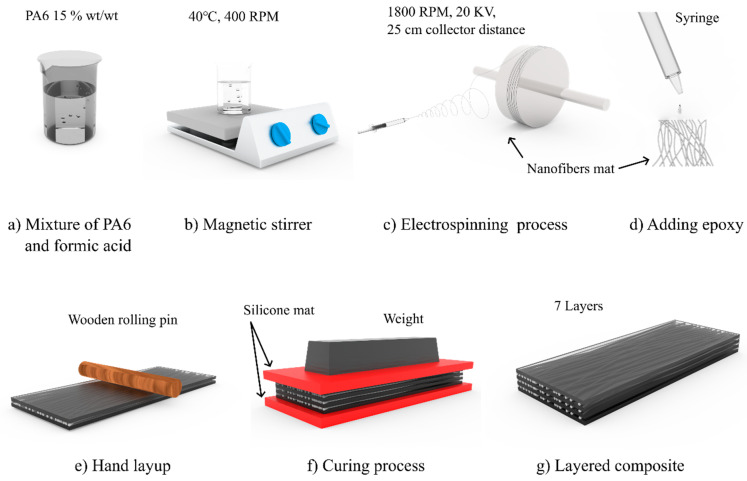
The fabrication process of the layered composite. (**a**) preparation of PA6 granules and formic acid mixture; (**b**) Magnetic stirrer of mixture at 40 °C at 400 RPM; (**c**) Fabrication of electrospun nanofibers; (**d**) Appling epoxy to nanofiber mat (**e**) Hand layup to avoid voids between the layers; (**f**) epoxy curing process under the pressure between the silicone mat; (**g**) layered nanocomposite.

The polymer polyamide solution was prepared by adding the PA6 granules to the solvent (formic acid) at 15% wt/wt and mixing for 6 h with a magnetic stirrer (Thermo Scientific™ Cimarec + ™ Stirring Hotplates Series, USA) under +40 °C (the room temperature was +22 ± 1 °C; humidity, 60%) and a stirring speed of 400 rpm (Figure 1a,b). In order to eliminate the air bubbles and stabilize the solution, it was left at room temperature for 1 h.

The PA6 nanofibers were spun (Figure 1c) at a room temperature of +22 ± 1 ℃, using an electrospinning setup: Fisherbrand™ Single Syringe Pump, a needle-based electrospinning machine, Danbury, (CT 06811), USA, and a Rotating Collector RC-5000, Diameter 140 mm, Length 50 mm, Shenzhen Tongli Tech Co Ltd., (D-608) Shenzhen, China. A one-mL plastic syringe and needle, type 23 Ga, were used. The electrospinning parameters used in our work were a voltage of 20 kV and a flow rate of 0.6 mL/h, and the distance between the syringe type and collector drum center was 25 cm. The rotating speed of the drum collector was constant at 1800 rpm (tangential speed = ~13.2 m/s), and aluminum foil (width 10 cm and thickness 35 µm; Vireo.de, Merseburg, (06217) Germany)) was used on the drum to collect the nanofibers. For mechanical properties’ analysis, the specimens were collected after 1 h of electrospinning. A scanning electron microscope (SEM, Hitachi High-Tech TM Series TM3030 Plus scanning electron microscope (The Netherlands)) was used for the specimens’ morphology analysis; the specimens were obtained after 30 min of electrospinning. Before any studies or characterizations were performed, all specimens were kept at a room temperature of +22 ± 1 °C and relative humidity less than 60% for a period of 48 h (according to ISO 139:1973, Textiles—Standard atmospheres for conditioning and testing).

### 2.3. Fabrication of the Multilayered Nanocomposites

Figure 1 depicts the whole process of creating the layered nanocomposites. The ratio of epoxy resin to hardener was 10:1 wt./wt.% at a room temperature of +22 ± 1 °C and a relative humidity below 60%. The epoxy hardener was loaded into a 1 mL syringe, and a drop (0.05 ± 0.001 mL) was spread over a 60 mm × 60 mm region of a silicone mat using a wooden rolling pin (Figure 1d,e). As previously described, a nanofiber mat was put on top of the applied epoxy, and more epoxy was applied to the nanofiber mat. As seen in Figure 1, this procedure was performed three times to create seven layers of the layered composite (Figure 1g). For 48 h, a second silicone mat was placed on the created layered sample and pressed down with a ~654 N/m^2^ until it solidified (Figure 1f). After the specimen cured, the edges were trimmed, and the center of the composite was exactly cut to 10 mm × 50 mm; the nanofiber alignment remained along the longitudinal axis throughout the process. 

### 2.4. Morphology of the Spun Nanofiber and Layered Composite

The SEM images were taken with a TM300 Tabletop Microscope SEM (Hitachi) that had a magnification of 1500, a vacuum of 10-2 Torr, an ion coater that used 6 mA, a gold (Au) cover, and a coating thickness of 10 nm. The fiber orientation was obtained using the ImageJ software’s OrientationJ plug-in [43,44] (ImageJ, National Institutes of Health, Bethesda, MD, USA). The average nanofiber diameter and standard deviation were determined by measuring the diameter of 300 nanofibers randomly chosen from three SEM images. The thickness of the nanocomposites was determined by a digital micrometer QuantuMike (Mitutoyo 293-185-30 QuantuMike Micrometer Digimatic 0-1 Inch Ip65.5 No Spc KB364, Japan, computer personal calibration at start-up), with a discreteness of 0.001 mm; the thickness measurements of each specimen were taken at three different points.

### 2.5. Tensile Properties 

**Figure 2:**
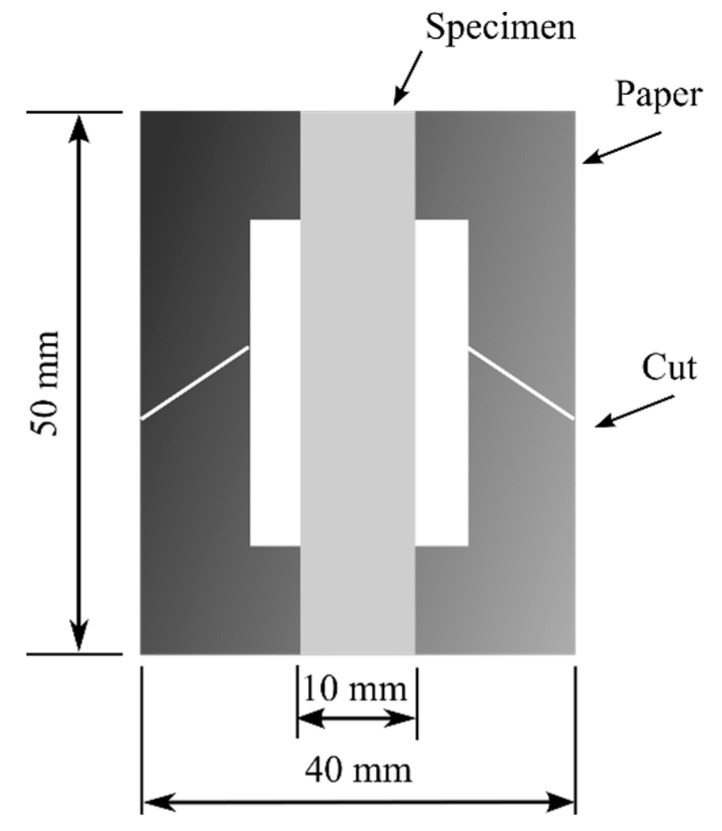
Specimen with piece of paper.

Mecmesin’s Multi-Test 2.5-i tensile testing machine (PPT Group UK Ltd., t/a Mecmesin, Newton House, Spring Copse Business Park, Slinfold, West Sussex RH13 0SZ, United Kingdom) was used to measure the tensile properties. The *x*-axis speed was 5 mm/min, and the testing conditions, according to the ISO 139:1973, Textiles—Standard atmospheres for conditioning and testing, were a temperature of +21 ± 1 °C, a relative air humidity of 60%, and an atmospheric pressure of 760 mm Hg. The size of the samples was 50 × 10 mm (length and width). Ten measurements were conducted in order to determine the tensile characteristics. Using a digital micrometer, the thickness of the test specimens was measured. The thickness of the specimen was the mean of the thicknesses measured at three different points. The specimen was cut parallel to the nanofibers’ direction. Figure 2 depicts the specimen with a papercut frame that facilitated the specimen mounting on the Mecmesin’s Multi-Test 2.5-i tensile testing machine. A 50 mm × 40 mm paper frame with an inside cut of 30 mm × 20 mm was created. Both ends of the specimen were adhered to the paper frame using double-sided thin Scotch tape (3M Scotch Magic Tape (Matte Finish) 3/4”x36 yards Desk Dispenser Refills). After attaching the paper frame and specimen to the tensile testing machine, the sides of the paper frame were cut off using scissors. In the same way, the nanofiber mat was tested on a 25 N sensor that was 40 mm × 10 mm and had a paper frame that was 40 mm × 40 mm with a 20 mm × 20 mm opening in the middle. The equivalent thickness of the nanofiber mats was determined by the specimen’s area and the average mass (using laboratory scales KERN ABT 5NM (KERN&Sohn GmbH; D-72336, Balingen, Germany); max till 100 g, discreteness 0.00001 g; calibration certificate number: M0901K22, 15.06.2022) with the density of the pure polyamide (PA6) [45]. To test the tensile strength of the nanofiber mats, 10 specimens were made. Five of these were used to figure out the mass and equivalent thickness based on the mass, and the other five were used for tensile testing with the same thickness. 

For statistical significance, the observed tensile Young’s modulus was averaged across at least ten specimens. The elastic modulus of the nanocomposites was also evaluated using the fundamental rule of mixing (ROM) for comparison purposes.

(1) $$E_{C}={\;E}_{E}{\;V}_{E}+{\;E}_{\mathrm{PA}6}\;\left(1-{\;V}_{E}\right),$$

where E_C_ is the nanocomposite’s Young’s modulus, E_E_ and E_PA6_ are the experimental Young’s modulus for the epoxy and nanofiber mat, respectively. The E_C_’s value forecasts the linear relation between E_C_ and the nanofiber mat. In addition, the reinforcing effectiveness of the PA6 nanofiber mat compositions was calculated numerically.

Similarly, the Young’s modulus was calculated assuming a random in-plane fibers orientation using the Tsai–Pagano model in Equation (2) [46].

(2) $$E_{C}=\frac{3}{8}{\;E}_{L}+\frac{5}{8}{\;E}_{T}$$

(3) $$E_{L}=E_{\mathrm{PA}6}V_{\mathrm{PA}6}+E_{E}\left(1-V_{\mathrm{PA}6}\right)$$

(4) $$E_{T}=\frac{E_{\mathrm{PA}6}E_{E}}{E_{\mathrm{PA}6}\left(1-V_{\mathrm{PA}6}\right)+E_{\mathrm{PA}6}V_{E}},\;$$

where E_L_ and E_T_ are the longitudinal and transverse modulus of the nanocomposite, respectively, computed longitudinally and transversely to the direction of the fibers, assuming a unidirectional composite with the cylinder fibers.

**Figure 3:**
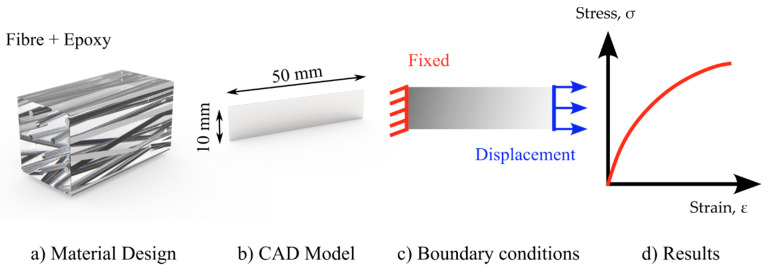
Procedure of the numerical simulation; (**a**) Material design of fiber reinforced epoxy (**b**) CAD model with the size of tested specimen (**c**) Applied boundary conditions to the model, one end fixed and displacement at another end (**d**) Stress-Strain graph obtained as a results.

Figure 3 depicts the numerical simulation procedure. In ANSYS, the material design was utilized to construct a material for numerical simulation. The testing results of the PA6 mat and pure epoxy were included in the material model (Figure 3a), with the parameters of all fibers randomized within a 15-degree range relative to the loading axis. The material design was based on the assumption that the random unidirectional fiber composites were made up of an isotropic linear-elastic matrix material and an isotropic or transversely isotropic (in one direction) linear-elastic fiber material. Each fiber had the same diameter and was cylindrical in form. There was perfect attachment between the fibers and the matrix material. On average, the fibers were oriented in one direction (along the loading axis). The material design was applied to a 50 × 10 mm CAD model Figure 3b (the same as the tested specimens).

For the boundary conditions, one end of the geometry was fixed, while the other was allowed to move by 1 mm. The acquired findings of the force and elongation were compared to the experimental data (Figure 3c,d). 

### 2.6. Thermal Properties

Differential scanning calorimetry (DSC) investigations were conducted using a DSC 214 Polyma (Netzsch, an der Universität 2, (30823) Garbsen, Germany) under a nitrogen (N_2_, CID 947) environment with a 30 mL/min flow rate. The pure PA6 and layered composite were cut accurately, put in the crucible, and held for 48 h at a temperature of +22 ± 1 °C and relative humidity of less than 60%. The samples were heated at a rate of 15 K/min from −40 °C to +230 °C.

## 3. Results and Discussion

### 3.1. Morphology 

**Figure 4:**
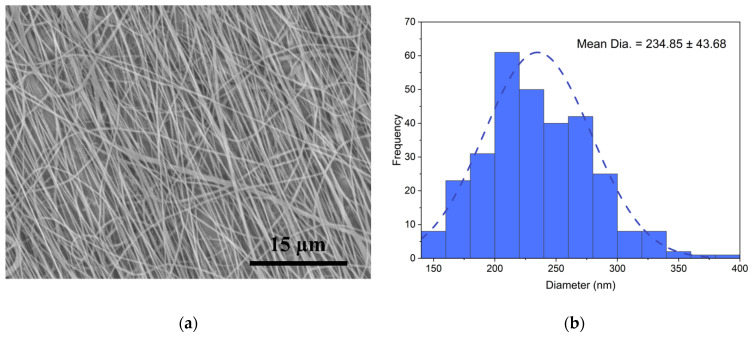
(**a**) Morphology of the PA6 nanofibers. (**b**) Diameter distribution of the PA6 nanofibers.

Figure 4a depicts the SEM image of the PA6 nanofibers utilized for FFT analysis and nanofiber alignment observation. Image processing was performed, and the directional distribution of the fibers was evident, which facilitated the FFT analysis technique. Figure 3b depicts the diameter distribution graphs for the nanofibers. The skew of the specimen diameter distribution of the nanofibers collected at various speeds (|A|) was less than 0.5; hence, Gaussian distribution could be used. The nanofibers had an average diameter of 234.85 ± 43.68 nm.

**Figure 5:**
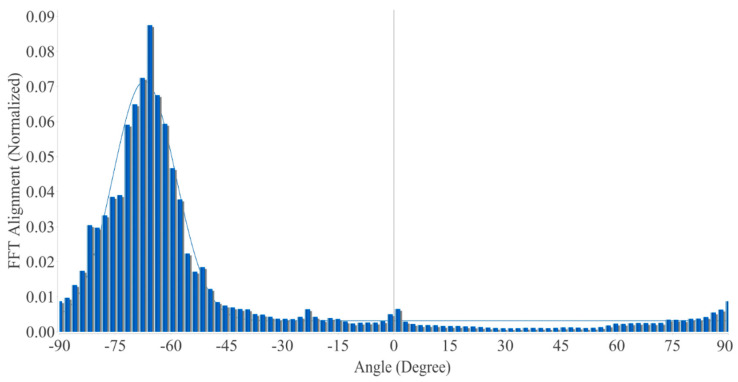
Alignment of the PA6 nanofibers.

Figure 5 shows the results of an investigation into the alignment of the PA6 nanofibers used to fabricate the multilayer composites. The findings of the investigation indicated that the FFT Alignment (normalized) value ranged from 0.0 to 0.09, while the drum rotated at 1800 rpm. Most of the nanofibers were oriented in one direction. Increasing the rotation speed of the drum collector at a steady rate stabilized the drawing process and enabled the achievement of a uniform diameter range [47].

**Figure 6:**
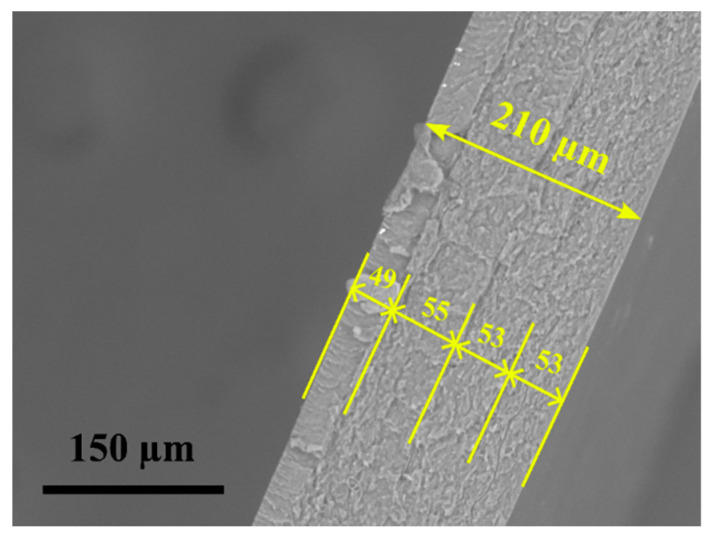
SEM image of the nanocomposite.

The layered nanocomposite SEM image is seen in Figure 6. The PA6 and epoxy nanocomposite films handcrafted layup exhibited excellent layer distribution, as seen by the nanocomposite’s consistent translucency. Even in the epoxy layer, there were no visible traces of voids or agglomeration, demonstrating a good layered morphology. The composites were usually flat and easy to cut into rectangular test specimens. The average thickness was 211.1 ± 5 µm, and the thickness of each epoxy layer was 52.5 ± 3 µm. 

### 3.2. Tensile Tests

**Figure 7:**
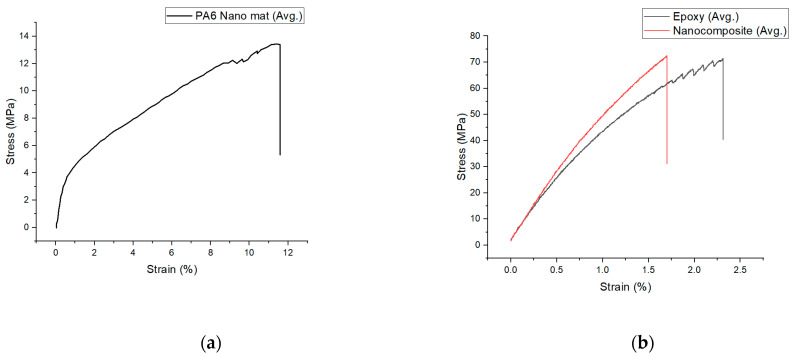
Stress–strain curve. (**a**) PA6 nanofiber mat; (**b**) epoxy and layered composite.

Figure 7 and Table 1 depict the stress (σ)–strain(ε) graphs of the PA6 nanofiber mats, epoxy, and layered composite. There were no failures at the grips during tensile testing due to the clamping pressure. It is evident from the force–displacement graphs of the epoxy and nanocomposites that the strength of the nanocomposites was higher than that of the pure epoxy after adding nanofiber mats. The nanofiber mat has a tensile strength of 13.18 ± 1.54 MPa and a Young’s modulus of 3200 ± 15 MPa. It was established in this study that the addition of the well-aligned nanofibers (mats) to an epoxy matrix enhanced the tensile strength from 74.45 ± 3.50 MPa to 76.84 ± 4.74 MPa and the elastic modulus from 2070 ± 10 MPa to 2315 ± 19 MPa.

The increase in the tensile strength and Young’s modulus of the nanocomposite is dependent on the strength of the reinforced nanofibers [37]. In this research, the modest increase in strength was entirely dependent on the strength of the nanofiber mat and its structure. An important aspect influencing the mechanical characteristics of the nanofiber mats is anisotropy. When the nanofibers are oriented randomly, the strength of the mat is less than when the nanofibers are oriented in one direction. In addition, when the drum collector’s rotation speed is more than 1000 rpm, the resulting nanofibers have a more aligned orientation [48,49]. The orientation of the nanofibers enhances the nanofiber mats’ tensile strength. Much research has shown that the nanofiber mats collected above 1000 rpm have superior mechanical qualities compared to those collected below 1000 rpm or on a flat plate collector.

In addition, it has been discovered that the Young’s modulus and tensile strength of the nanofibers improve as their diameter lowers, particularly when the diameter drops below 700 nm. Studies based on nanofibers have revealed that the mechanical characteristics grow exponentially as the fiber diameter drops to a critical point. When the nanofibers are intertwined, the nanofiber’s structure has fewer irregularities and a more uniform structure, resulting in increased endurance [21,50,51,52,53]. In our research, we found that when nanofibers were collected at 1800 rpm with an average diameter of 234.85 ± 43.68 nm, the young’s modulus was 3200 ± 15, which was higher than when the nanofibers were collected at a lower speed or on a flat plate.

The tensile stress of the nanofiber mats is influenced by several variables, including the chemical structure of the polymer, its molecular orientation, and its extension chain. The random or ordered distribution of the amorphous and crystalline phase controls the nanofibers’ physical and mechanical characteristics [54,55]. In the electrospinning process, the polymer molecules are pushed apart by electromagnetic forces, resulting in the orientation of the polymer molecules. Using a revolving drum collector with a faster rotational speed resulted in a more targeted mechanical force on the polymer molecule. This strengthened the durability of the nanofiber mats. The alignment of the nanofibers in the mats, the reduction in fiber diameter, and the molecular orientation phase of the polymer were responsible for the rise in the elastic modulus, which correlated with the results shown in [41,55]. Prior research has demonstrated that the addition of the nanofiber mats as laminates or in a mixture of the polymeric solution to epoxy-based composites improves the nanocomposites’ mechanical performance [9,56,57,58,59]. In addition, heat is produced during the curing phase of the epoxy resin, which enhances the strength of nanofiber mats by up to 45% [60]. In summary, the rise in the elastic modulus in the nanocomposite was a result of the adding the nanofiber mat.

**Figure 8:**
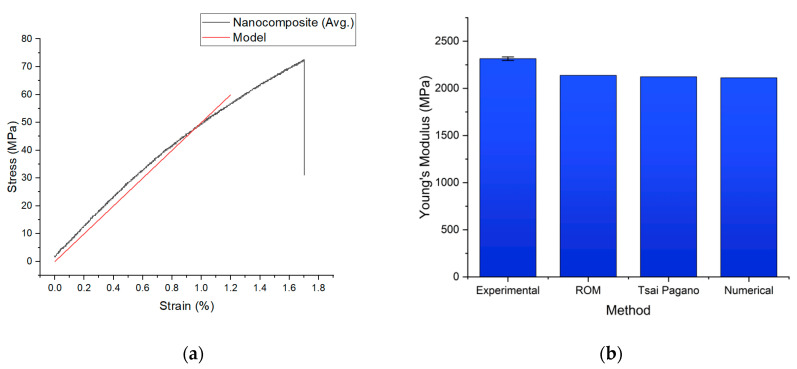
Data comparison for the nanocomposites. (**a**) Experimental and numerical simulation stress–strain graph; (**b**) comparison of the Young’s modulus.

Figure 8 compares the numerical simulation and the experimental findings of the composites’ elastic regions. According to the numerical simulation, the composite Young’s modulus was 2112.5 MPa, which was slightly less than the analytically predicted value with the ROM of 2137.8 MPa (based on Equation (1)) and 2123 MPa (based on Equation (2)), based on the prediction of the Tsai–Pagano model. The experimental Young’s modulus value of 2315 ± 19 MPa was higher than the theoretical value.

As a reasonable approximation of the elastic modulus (Ec) of the nanocomposites, we used the fundamental rule of mixtures model (ROM). The ROM assumes that Ec is proportional to the strength of the PA6 nanofibers and the epoxy, scaled to the fiber volume percentages. Assuming that the tensile strength of a single nanofiber is underused, Equation (1) predicts that the nanocomposites will possess a maximum tensile strength. The difference in tensile strength is a result of the nanofibers and nanofiber networks [61]. The observed values of the tensile strength nearly matched the projected values for the composition formulations, as predicted by the ROM model. Comparing the observed elastic moduli for the nanocomposites with the moduli computed using Equation (2), the Tsai–Pagano model underestimated the nanocomposites’ elastic moduli.

### 3.3. DSC Analysis

**Figure 9:**
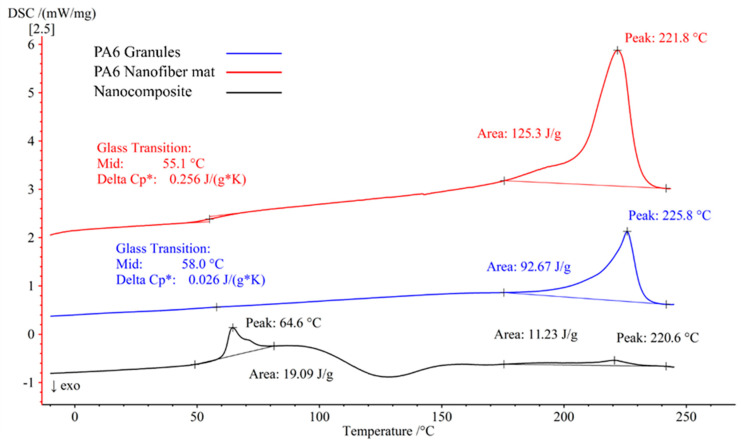
Differential scanning calorimetry (DSC) of the PA6 granules, PA6 nanofiber mats, and the nanocomposites.

The DSC was used to examine the polymer’s melting and crystallization behavior in the PA6 pure, PA6 nanofiber mat, and the nanocomposite. Figure 9 shows the DSC analysis result. The PA6 granules had a glass transition temperature (Tg) of +58.0 °C and a melting temperature (Tm) of +225.8 °C, whereas the PA6 electrospun nanofiber mats had a Tg of +61.0 °C and a Tm of +221.8 °C. Due to the layered epoxy in the nanocomposite, the epoxy’s glass transition was observed at +64.6 °C, whereas the melting caused a second peak in the graph at +220.6 °C. The nanocomposites had a higher temperature of glass transition than the PA6 granules and PA6 electrospun nanofiber mats. An increment in the glass transition temperature may be related to a reduction in the diameter of the fibers relative to the volumetric dimensions. At glass transition, the specific heat capacities (Delta Cp*) of PA6 granules and PA6 nanofiber mat were 0.026 J/(g*K) and 0.256 J/(g*K), respectively, with the nanofiber mat having about 10 times the specific heat capacity. Table 2 displays the transition temperatures of the pure PA6 granules, nanofiber mat, and the nanocomposite.

In Figure 9, the DSC results for the first heating cycles are shown. There was a moderate morphological change in the crystal structure of the polymer between the PA6 granules and the PA6 nanofiber mat, as shown by the 4 °C shift in Tm found in this study, which corresponded to a decrease in the crystalline order of the nanofiber mat crystals [62]. This shift in the crystallinity indicated that the evaporation of the formic acid produced a metastable crystalline structure [63]. The Tg of the nanofiber mat was somewhat lower than that of the PA6 granules. This decrease in the Tg was due to the uniform distribution of the nanofibers [64]. 

### 3.4. Structure and Morphology after Tensile Testing

**Figure 10:**
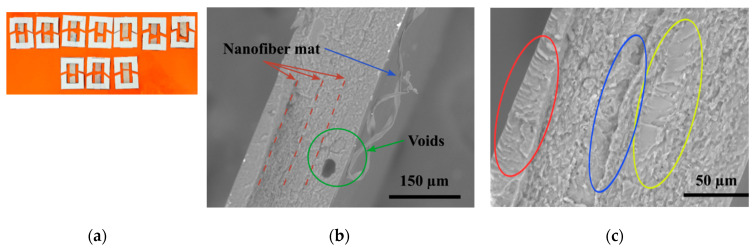
Tested specimens of the nanocomposites: (**a**) breakage points of the specimens; (**b**) SEM image of a broken specimen; (**c**) tensile fracture surface.

After the material has been strained enough, the middle will become thinner and weaker, so it should break in the middle. Figure 10a depicts the specimen of the nanocomposites subjected to testing; all specimens broke from the center area, but none were damaged near the clamps. In the epoxy layer, the crack inclined toward the crack propagation direction, and due to the formation of a fracture in the epoxy layer, the nanocomposite failed, as shown in the SEM in Figure 10b. The nanofiber mat was detached when the epoxy coating failed which corresponds to similar result found in carbon/epoxy laminates the nanofibers seem to have been peeled off from the matrix resin without much deformation [16]. In addition, a void (air trap) was discovered at the point of failure, which was not spotted on the composite’s exterior surface.

Figure 10c shows the SEM picture of the specimen failure. The precise bonding between the PA6 nanofiber mat and the epoxy can be seen (blue zone), and it is evident that the PA6 layer adhered flawlessly to the epoxy. The crack grew gradually and produced a smooth surface (yellow region). The fracture accelerated and generated a rough patch; when the crack achieved its maximum speed, a very rough region developed (red region). This was consistent with the findings of the tensile test. The composite’s strength was increased as a result of the bridging effect [16] at the interface of the PA6 nanofiber mat and epoxy layer, which increased the crosslink intensity and dissolution bonds [65,66]. 

## 4. Conclusions

The hand layup method was used to make epoxy nanocomposites that were reinforced with a PA6 nanofiber mat. Due to the high interface adhesion between the epoxy and the PA6 nanofiber mat, the nanocomposite formed from the epoxy and nanofiber reinforcing effect allowed for an effective stress transmission, resulting in good dispersion. The tensile test findings revealed an outstanding reinforcing effect, as the nanocomposites’ tensile moduli increased by 10.58%. With three layers of nanofiber mat, the highest elastic modulus value of 2315.5 ± 19 and tensile strength of 76.84 ± 4.74 were achieved. The uniformity of the nanocomposite films indicated an excellent coating of epoxy and the PA6 nanofiber mat. In addition, it demonstrated the significance of the nanofiber diameter and orientation on the nanocomposites’ strength. The DSC demonstrated increases in the glass transition temperature of the nanocomposite and the epoxy layer melted later around +220.6 ℃. This work proposed a technique for the development of lightweight high-performance nanocomposites.

## Figures and Tables

**Table 1:** Mechanical properties of the PA6 nanofiber mat, epoxy, and layered composite.

Materials	Tensile Strength at Break, σ (MPa)	Young’s Modulus, E (MPa)	Elongation at Break, ε (%)
PA6 nanofiber mat	13.18 ± 1.54	3200 ± 15	11.240 ± 1.01
Epoxy	74.45 ± 3.50	2070.4 ± 10	2.320 ± 0.24
Nanocomposite	76.84 ± 4.74	2315.5 ± 19	1.628 ± 0.3

**Table 2:** The thermal properties of the PA6 granules, electrospun PA6 nanofibers, and the nanocomposites.

Materials	Tg (°C)	Tm (°C)	h (J/g)
PA6 granules	58.0	225.8	92.67
PA6 nanofiber mat	55.1	221.8	125.3
Nanocomposite	64.6	220.6	19.09/11.23

## Data Availability

All data are provided in the paper.
